# Supplementary material for: Genome-wide Identification of conditionally essential genes supporting Streptococcus suis growth in serum and cerebrospinal fluid
Source: Virulence. 2025 Dec 15;17(1):2600145. doi: 10.1080/21505594.2025.2600145 (PMC12707516; doi:10.1080/21505594.2025.2600145)
Supplement: Table S1.docx [file KVIR_A_2600145_SM0358.docx]

### Table S1.Stock materials for CDM

| Composition of CDM buffer in 100 ml water | |
| --- | --- |
| Na-B-glycerophosphate | 9.3 g |
| KH_2_PO_4_ | 0.4 g |
| (NH_4_)_2_ citrate | 0.25 g |
| Na acetate | 0.44 g |
| **Adjust pH to 6.4* | |
| Bacto tryptone | 7.5 g |
| Metal mixture in 10 ml water | |
| MgCl_2_ | 0.103 g |
| CaCl_2._2H_2_O | 54 mg |
| ZnSO_4_.7H_2_O | 5.5 mg |
| CoSO_4_.7H_2_O | 3.3 mg |
| CuSO_4_.5H_2_O | 0.17 mg |
| Vitamin mixture in 10 ml water | |
| Pyridoxal-Cl | 2 mg |
| Thiamine Cl_2_ | 1 mg |
| Riboflavin | 1 mg |
| Ca-pantothenate | 1 mg |
| Biotin | 0.1 mg |
| Folic acid | 1 mg |
| Vitamin B6 | 1 mg |
| **Light sensitive reagents, adjust pH to 7* |  |
| Amino acids | **Concentration (g/L)** |
| Alanine | 3 |
| Glycine | 3 |
| Arginine | 3 |
| Serine | 3 |
| Threonine | 6 |
| Cysteine | 3 |
| Proline | 3 |
| Asparagine | 3 |
| Aspartate | 3 |
| Methionine | 3 |
| Lysine | 3 |
| Glutamine | 6 |
| Histidine | 3 |
| Glutamate | 3 |
| Phenylalanine | 3 |
| Tryptophan | 3 |
| Valine | 3 |
| Leucine | 3 |
| Isoleucine | 3 |
| Additional stock material | **Concentration** |
| MnSO_4_.H_2_O | 28 mg/ml |
| Choline chloride | 2.5 mg/ml |
| Pyruvate | 1 mg/ml |
| Glucose | 50 M |
| Nucleotide/ nucleoside | **Concentration (mg/ml)** |
| Adenine | 1 |
| Uracil | 1 |
| Thymidine | 1 |
| Cytidine | 1 |
| Guanine | 1 |
