# Supplementary material for: Genome-wide Identification of conditionally essential genes supporting Streptococcus suis growth in serum and cerebrospinal fluid
Source: Virulence. 2025 Dec 15;17(1):2600145. doi: 10.1080/21505594.2025.2600145 (PMC12707516; doi:10.1080/21505594.2025.2600145)
Supplement: Revisions_data.docx [file KVIR_A_2600145_SM0355.docx]

**Figure 1**. Growth of S. suis WT, ΔpurA, ΔhdrM, and ΔSSU_RS04755 (reconstructed with non-overlapping CRISPR guides) in adult porcine serum (APS; top, CFU/mL; y-axis log₁₀) and THY (bottom, OD₆₀₀; y-axis log₁₀) at 37 °C over time. In APS, ΔpurA and ΔSSU_RS09155 reach lower CFU/mL than WT, while ΔSSU_RS04755 is close to WT. In THY, all strains show similar OD₆₀₀ profiles. Points show mean ± SD of 3 biological replicates.


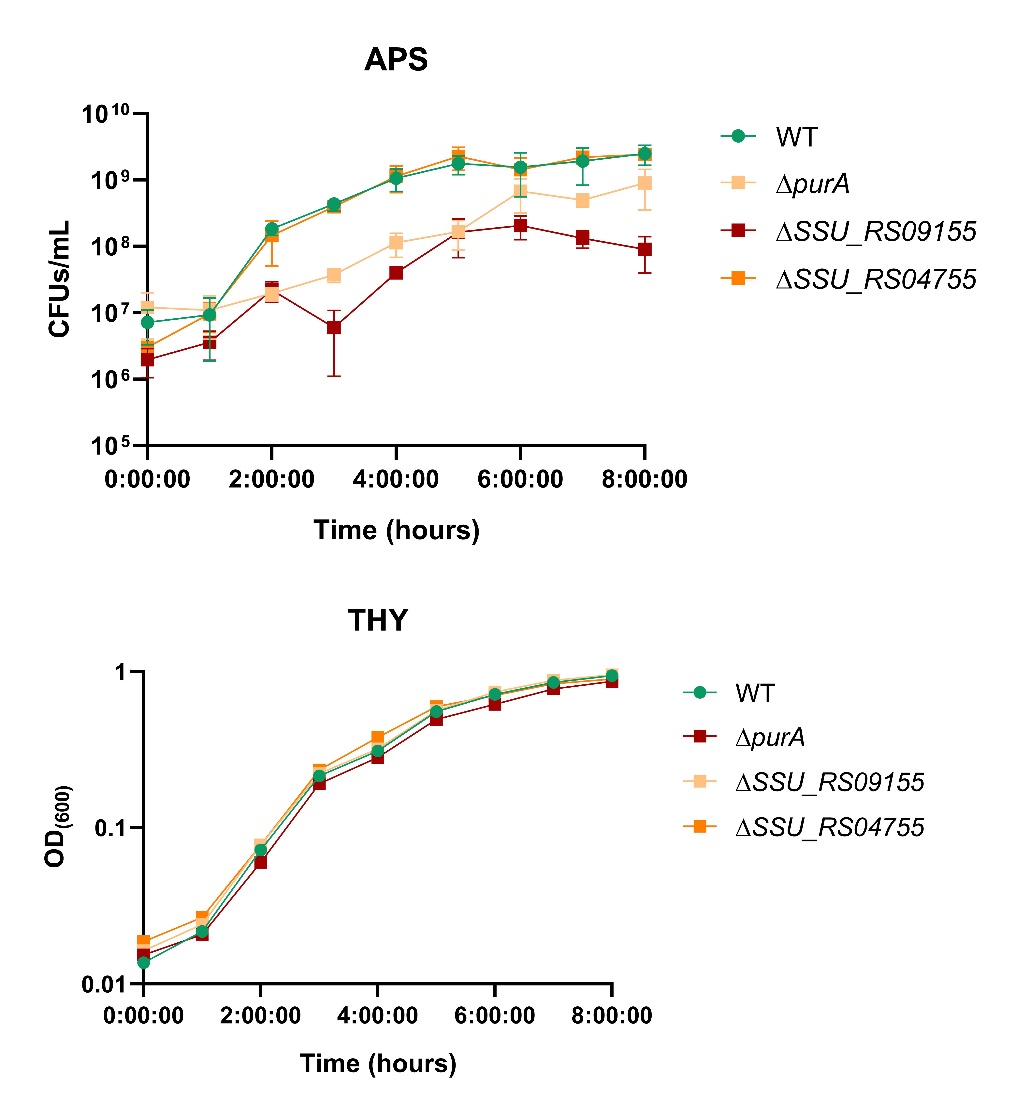


**Table 1.** List of the new guides employed to regenerate the ΔpurA, ΔhdrM, and ΔSSU_RS04755 mutants used in the validation assays. Phenotypic outcomes correspond to Figure 1.

| **Name** | **Sequence** |
| --- | --- |
| *purA* gRNA 3 FWD | **tgat**gcagccttgtccatgtaggc |
| *purA* gRNA 3 REV | **aaac**gcctacatggacaaggctgc |
| SSU_RS04755 gRNA 2 FWD | **tgat**taccagtaccaccagaagcg |
| SSU_RS04755 gRNA 2 REV | **aaac**cgcttctggtggtactggta |
| SSU_RS09155 gRNA 4 FWD | **tgat**ctcatggatgtatcgagtcg |
| SSU_RS09155 gRNA 4 REV | **aaac**cgactcgatacatccatgag |
